# Book Reviews

**Published:** 1916-01

**Authors:** 


### Book Reviews.

"Habits That Handicap."—The Century Co. of New York has recently issued a book on "Habits That Handicap." The book is the work of Charles B. Towns, whose life work has been the study and treatment of victims of habit-forming drugs. Mr. Towns is largely responsible for the legislative campaign through which we have just passed.

The introduction to the book is written by Richard C. Cabot, who has sent Mr. Towns numerous patients, all of whom were quickly and effectually rid of the morphia addition, and he says: "I do not hesitate to say that he knows more about alleviation and cure of drug addicts than any doctor that I have ever seen."

After the author had proven the efficacy of the treatment at home he decided to visit the Orient, where the drug habituates were more common than elsewhere. His desire to study this unfortunate class in large numbers led him to China, where he opened three hospitals, and in the course of eleven months supervised the treatment for the opium habit of four thousand Chinese. He relates that among the four thousand not one fatality occurred, though many of them were extreme cases. Indeed, he makes the statement that during his fourteen years of practice, he has lost only four patients.

The appendix is written by Dr. Alexander Lambert, Professor of Clinical Medicine in Cornell University. It is a short treatise on "The Relation of Alcohol to Disease." In this treatise the doctor states clearly the effect of alcohol upon the different members of the body, and defines very pointedly the difference between moderation and excess in the use of alcohol. The price of this book is $1.20.

---

The Starvation Treatment of Diabetes—With a Series of Graduated Diets as Used at the Massachusetts General Hospital. By Lewis Webb Hill, M. D., and Rena S. Eckman, Dietitian, with an introduction by Richard C. Cabot, M. D. Price, $1.00. W. M. Leonard, Publisher.

This book furnishes to the general practitioner in compact form the details of the latest and most successful treatment of diabetes mellitus. It presents the clinical application of the work done in recent years by Dr. Allen at Harvard and the Rockefeller Institute.

This method of treatment, carried out by Dr. Allen at the Rockefeller Hospital, and by the staff at the Massachusetts General Hospital, has proved very successful. As Dr. Cabot states in the introduction: "It seems already clearly proven that Dr. Allen has notably advanced our ability to combat the disease. * * * To all who wish to give their patients the benefit of this treatment, I can heartily recommend this book." In a recent address, Dr. Allen said: "However specialists may feel, there is no doubt that a majority of average practitioners feel bewildered and helpless concerning diabetes."

To all who have been tried by this baffling disease, this little volume, with its description of treatment, tests and diets will be of greatest service.

OPERATIVE GYNECOLOGY. By Harry Sturgeon Crossen, M. D., F. A. C. S., Associate in Gynecology, Washington University Medical School and Associate Gynecologist to the Barn's Hospital, etc. C. V. Mosby Company, 1915. Price, $7.50.

This volume is a new departure in medical literature, and for that reason commands special attention. It is devoted exclusively to gynecological operations, including the after-care and complications. Its special feature lies in the presentation and discussion of the operation best adapted to the special pathological condition involved. The best accepted techniques are given for the specific operation, and from the discussion following the operator is enable to select the one that best fits the case. This is particularly useful to the great number of men who assay to do surgery but whose opportunity for seeing a very great variety of conditions is limited. To such this volume affords a judicious consultant.

The illustrations are numerous and are plain enough to illustrate the technique without reference to the text. No condition, however small, requiring surgical procedure, is omitted, including dysmenorrhea and other functional disturbances.

Enough pathology and symptomatology is given to clarify the discussions and make the volume a very practical guide that will be frequently consulted.

The concluding chapters deal with medico-legal questions and gynecologic surgery in nervous patients. The author's conclusions on this important question are timely and accords with the latest developments in physiology and neurology.

MALONE DUGGAN.

---

We have just received from the Year Book Publishers of Chicago Volumes VI and VII of the Practical Medicine Series.

Volume VI is edited by Frank Billings, M. D., who is the head of the Medical Department and Dean of the Faculty of the Rush Medical College of Chicago, and by J. H. Salisbury, M. D., who is Professor of Medicine of the Illinois Post-Graduate Medical School. Volume VI deals with infectious diseases, diseases of the mouth, stomach, intestines, liver and gall bladder and pancreas.

Volume VII is devoted to obstretrics and is divided into four parts. Part I deals with pregnancy. Part II deals with labor, and devotes quite a bit of space to twilight sleep. Part III deals with the puerperim, and Part IV deals with the new-born.

These books are well illustrated with cuts, charts and helpful drawings. Each book contains a complete index and shows an enormous amount of research work in the current literature of the day.

The volume on General Medicine sells for $1.35. The volume on Obstetrics sells for $1.50, and they are richly worth the price asked for them.

